# miR-145 promotes osteosarcoma growth by reducing expression of the transcription factor friend leukemia virus integration 1

**DOI:** 10.18632/oncotarget.9948

**Published:** 2016-06-11

**Authors:** Panfeng Wu, Jieyu Liang, Fang Yu, Zhengbing Zhou, Juyu Tang, Kanghua Li

**Affiliations:** ^1^ Department of Orthopedics, Xiang Ya Hospital Central South University, Changsha, Hunan, People's Republic of China

**Keywords:** miR-145, FLI-1, osteosarcoma

## Abstract

Osteosarcoma (OS) is the most common malignant bone tumor in children and young adults. miR-145 is a microRNA highly expressed in vascularized tissues and has been widely studied in cancers. In this study, we explored the expression and function of miR-145 in OS. We found that miR-145 was consistently under-expressed in OS tissues and cell lines as compared to normal bone tissues and osteoblast cells. Ectopic expression of miR-145 in OS cells inhibited their proliferation and migration and induced apoptosis. miR-145 targets a putative microRNA regulatory element (MRE) in the 3′-UTR of friend leukemia virus integration 1 gene (*FLI-1*), and its abundance was inversely related to *FLI-1* expression in OS tissues and cell lines. miR-145 decreased expression FLI-1 protein and mRNA, but mutation of the miR-145 MRE sequence in the *FLI-1* 3′-UTR abolished the activity of miR-145 in a reporter assay. Restored expression of *FLI-1* diminished miR-145-mediated suppression of tumor progression. These results suggest that miR-145 acts as a tumor suppressor by directly reducing expression of *FLI-1*, and that the miR-145/*FLI-1* pathway is important for tumor progression in OS.

## INTRODUCTION

Osteosarcoma (OS) is the most common bone tumor in children, representing 6% of all childhood cancers [1]. Although great efforts have been made to understand the underlying mechanisms of OS carcinogenesis, survival of OS has reached a plateau and the prognosis of advanced OS remains poor [2]. Therefore, novel diagnostic biomarkers and therapeutic targets for OS are needed.

MicroRNAs (miRNAs) are a class of small non-coding RNAs, which regulate gene function by targeting mRNA for translational repression or degradation [3]. miRNAs have been implicated in the pathogenesis of a variety of human diseases, including neoplasms [4]. Over-expression of oncogenic miRNAs or low expression of tumor suppressor miRNAs promote tumorigenesis [5]. A number of studies have reported an association between miRNA dysregulation and OS. In comparisons of miRNA expression profiles, miR-9, miR-18a, miR-145 and miR-451 were consistently decreased in both cell lines and clinical samples compared with normal bone tissues [6–8].

Among these miRNAs, miR-145 is a highly conserved gene among different species and is highly expressed in vascularized tissues such as liver, lung, and heart [9, 10]. Loss of miR-145 results in elevated tumor proliferation, migration, and survival, as well as increased leukocyte adhesion and disorganized tumor vasculature [11]. Recently, a cohort of genes related to cancer pathways have been identified and validated as targeted genes of miR-145, such as *P70S6K, C-MYC, PAI-1, FASCIN*, and *SOX-2* [12–16], suggesting that miR-145 is an oncogene that plays a pivotal role in the initiation and progression of cancer. However, the function of miR-145 in OS is largely unknown.

Friend leukemia virus integration 1 (*FLI-1*), a member of the ETS transcription factor family, is the target of insertional activation by the Friend murine leukemia virus and is preferentially expressed in vascular endothelial cells, embryonic tissue, and tumors [17, 18]. *FLI-1* plays a critical role in normal development, hematopoiesis, and oncogenesis by functioning either as a transcriptional activator or repressor [19–22]. Knocking down *FLI-1* expression in cancer cells leads to growth inhibition and cell death, demonstrating a possible therapeutic approach to induce tumor suppression [23, 24]. Anti-*FLI-1* compounds have demonstrated strong anti-leukemic activity in a mouse model that over-expresses *FLI-1*, making it possible to target *FLI-1* as an anti-tumor treatment [25]. However the role of the miR-145/*FLI-1* pathway has not been elucidated in osteosarcoma.

In this study, we explored the expression of miR-145 in 13 OS tumor tissues using PCR. We then completed a series of cellular functional experiments to investigate the role of miR-145 and its target genes in OS cell growth.

## RESULTS

### Down-regulation of *miR-145* and up-regulation of *FLI-1* expression in OS tissues and cell lines

**Figure 1:**
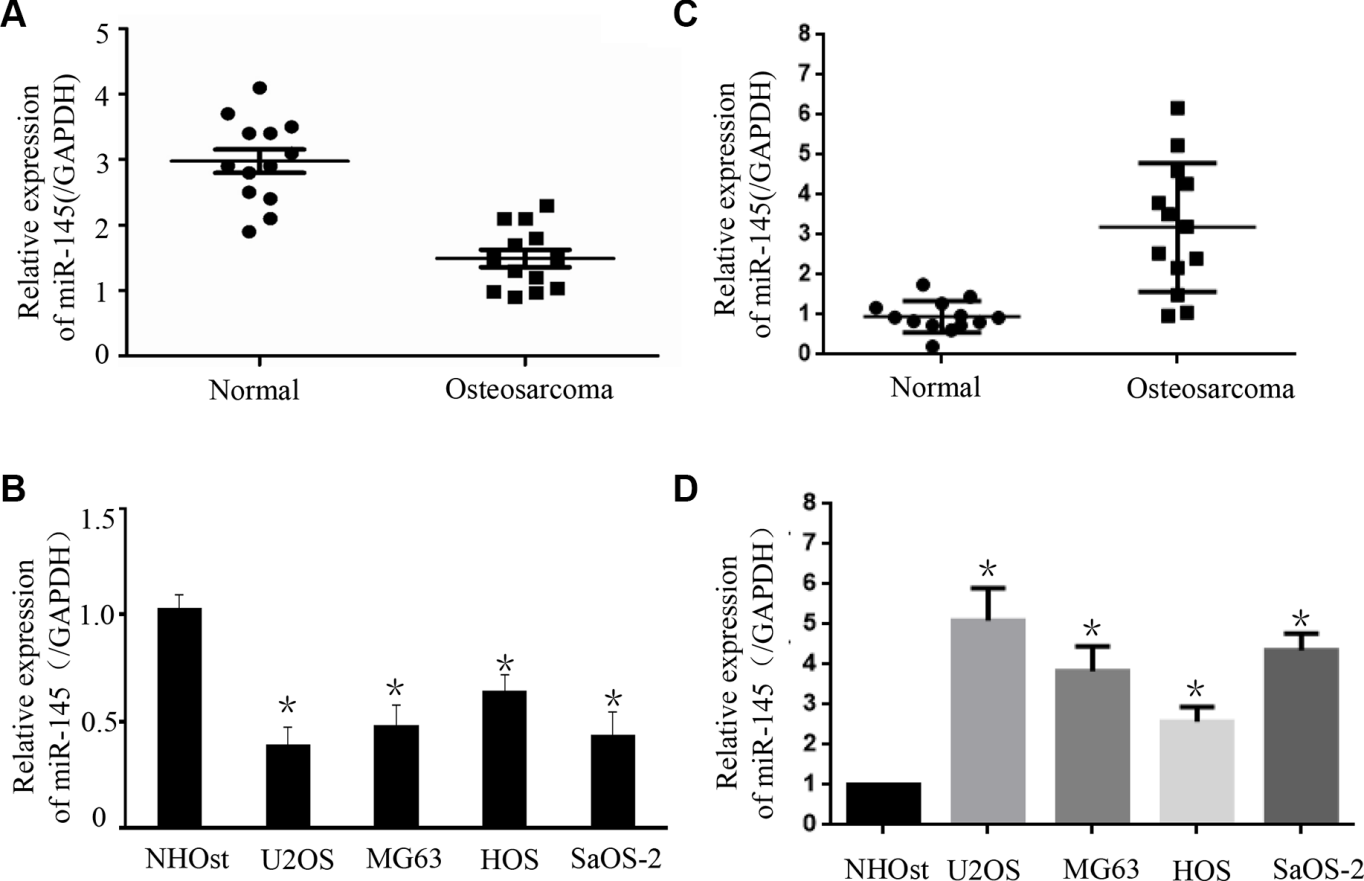
The expression of *miR-145* and *FLI-1* in osteosarcoma tissues and cell lines The expression of *miR-145* in 13 pairs of OS tissues and adjacent normal bone tissues was detected by TaqMan qRT-PCR. Data are shown as the ratio of OS tissues relative to normal bone tissues. (**A**) Statistical analysis of relative *miR-145* expression in OS tissues and compared normal tissues. Compared with normal bone tissues, the expression of *miR-145* in tumor tissues was down-regulated; and (**B**) Using qRT-PCR, the expression of *miR-145* in four OS cell lines (HOS, Saos-2, U2OS and MG-63) was analyzed. The expression of *miR-145* in these OS cell lines was down-regulated relative to normal osteoblast cells (NHOst). (**C**) Expression of *FLI-1* mRNA was analyzed in the OS tissues by qRT-PCR. (**D**) Using qRT-PCR, the expression of *FLI-1* mRNA in four OS cell lines (HOS, Saos-2, U2OS and MG-63) and one normal cell line was analyzed. *FLI-1* was up-regulated compared with the normal osteoblast cells (NHOst). *p < 0.01 compared with normal tissues or normal cells.

To explore the expression of *miR-145* and *FLI-1* in OS carcinogenesis, we examined 13 pairs of OS and matched normal tissues using TaqMan RT-PCR analysis. Relative to matched normal tissues, more than half of the OS tissues exhibited under-expression of *miR-145* mRNA and all of the OS tissues had high expression of *FLI-1* mRNA (Figure 1A & 1C). Expression of *miR-145* in OS tissues was lower than in normal tissues (Figure 1C). We next explored the expression of *miR-145* and *FLI-1* in four OS cell lines (HOS, Saos-2, U2OS, and MG-63). Compared with the normal human osteoblast cell line (NHOst), miR-145 expression was reduced in the four OS cell lines (Figure 1B). Interestingly, expression of *FLI-1* mRNA in all of the four OS cell lines was higher than in the NHOst cells (Figure 1D). These results suggested that the under-expression of *miR-145* and over-expression of *FLI-1* mRNA are common features of OS tissues and cell lines.

### miR-145 suppresses OS cell growth

**Figure 2:**
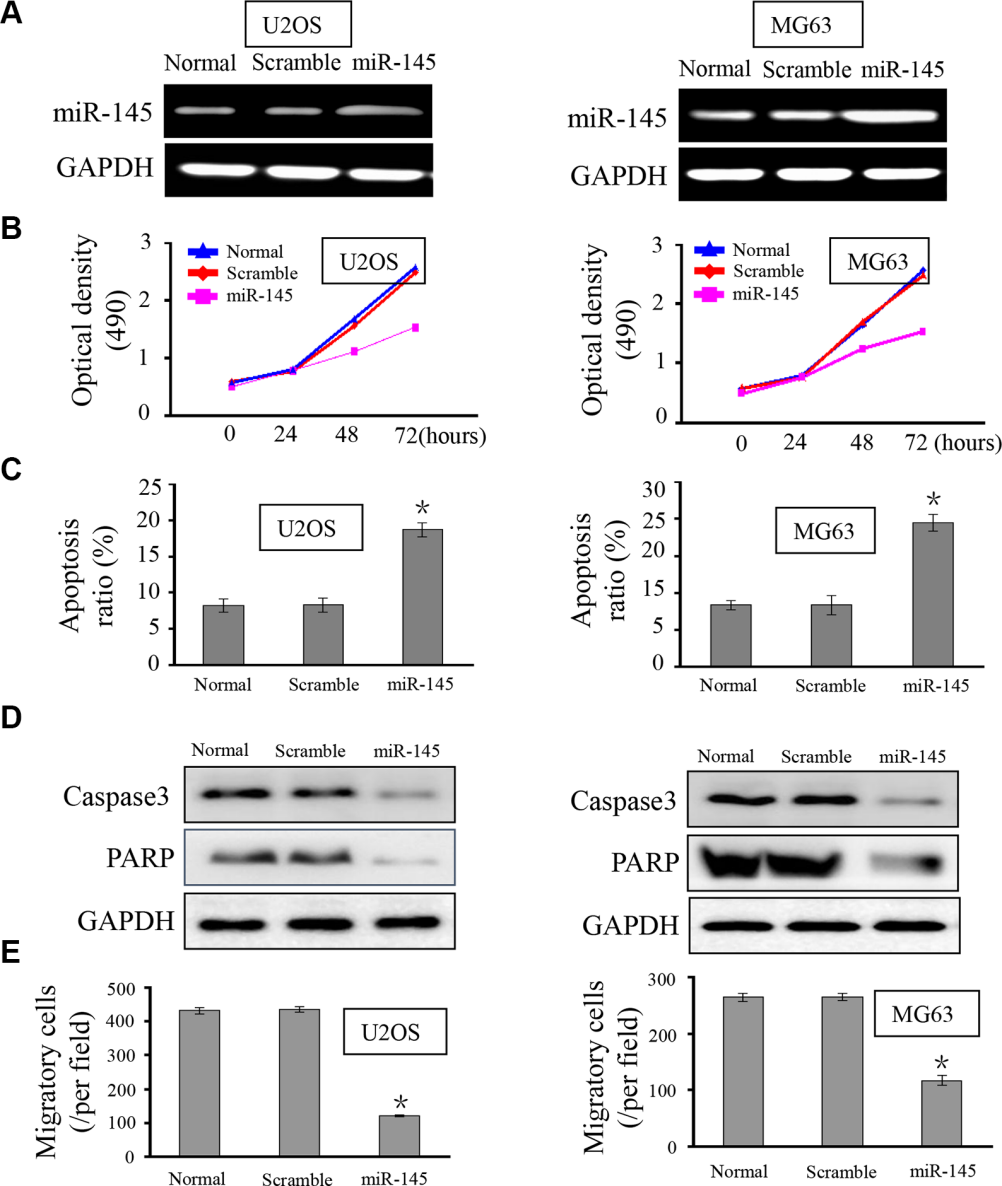
miR-145 suppresses OS cell proliferation, apoptosis and migration (**A**) RT-PCR was performed to detect the expression of *miR-145* in OS cell lines U2OS and MG-63. Upon transfection with the miR-145 mimic, expression of *miR-145* in U2OS and MG-63 cells was restored; (**B**) CCK-8 assay was performed to analyze the effect of miR-145 on U2OS and MG-63 cell proliferation. Ectopic expression of miR-145 inhibited cell proliferation; (**C**) The influence of miR-145 on cell apoptosis was analyzed. Transfection with miR-145 revealed more cells undergoing early apoptosis; (**D**) Caspase 3 and PARP expression were assayed by western blot in the OS cell lines; (**E**) The effects of miR-145 on cell migration were detected using transwell assays. Over-expression of miR-145 dampened cell migratory capacity; *p < 0.001 compared with scramble group.

The under-expression of *miR-145* in both human OS tissues and OS cell lines prompted us to explore its possible biological role in OS carcinogenesis. We sought to compensate for the reduced expression of *miR-145* by transfecting MG-63 and U2OS cells with miR-145 mimic. The intracellular level of miR-145 was about 4-fold higher in U2OS and MG-63 cells transfected with the miR-145 mimic relative to the scramble control group (Figure 2A). Cell proliferation was measured using CCK-8 assays. Ectopic expression of miR-145 led to a decrease in cell proliferation in both OS cell lines (Figure 2B). As proliferation directly links to cell apoptosis, we next examined the effect of miR-145 on apoptosis. As expected, the percentage of apoptotic cells was increased in both OS cell lines upon transfection with the miR-145 mimic (Figure 2C). To further understand the molecular mechanism of miR-145-induced apoptosis in OS cell lines we performed western blots. Ectopic expression of miR-145 reduced expression of apoptosis-related proteins, caspase3 and PARP (Figure 2D) in the two OS cell lines. Finally, given that migration promotes tumor metastasis, we investigated the effects of miR-145 on OS cell migration. As shown in Figure 2E, over-expression of miR-145 in OS cells reduced the number of migrating cells, suggesting a suppressive effect of miR-145 on OS cell migration. Taken together, these results indicated that miR-145 inhibits OS cell progression, and may function as a tumor suppressor.

### miR-145 regulates *FLI-1* expression in OS cells

**Figure 3:**
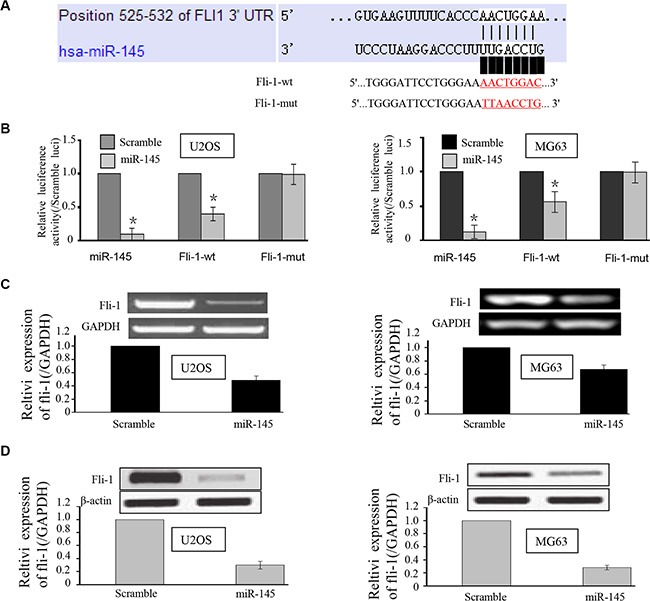
miR-145 targets the *FLI-1* gene in U2OS and MG-63 cells (**A**) Schematic representation of the *FLI-1* 3′UTR showing putative miRNA target sites. There was one putative binding site in the 3′UTR of miR-145; (**B**) Relative luciferase activity of the indicated *FLI-1* reporter constructs in U2OS and MG-63 cells, co-transfected with miR-145 mimics or scramble mimics. In cells co-transfected with pGL3-*FLI-1* 3′-UTR vector and miR-145 mimic, luciferase activity was suppressed relative to mutant construct groups; and (**C, D**) qRT-PCR and Western blot assays were performed to detect the expression of *FLI-1* upon transfection with miR-145 mimics or scramble mimics. Both mRNA and protein expression were suppressed upon transfection with miR-145. *p < 0.01 compared with scramble group.

In order to verify *FLI-1* as the target of miR-145, we used a luciferase reporter assay with plasmids containing the 3′UTR of human *FLI-1* co-transfected with miR-145. According to miRTarBase and targetscan, there is one validated binding site and two predicted binding sites for miR-145 in the *FLI-1* 3′UTR. In the current study, we only analyzed the validated binding site, as shown in Figure 3A. As expected, there was reduced luciferase activity in U2OS and MG-63 cells co-transfected with pGL3-*FLI-1* 3′-UTR vector and miR-145 mimic compared to cells transfected with the mutant construct (Mut-1, Figure 3B), suggesting that miR-145 suppresses the transcription of *FLI-1* by targeting binding sites in the 3′UTR. At 24 h post-transfection, western blot and RT-PCR analysis were performed. As shown in Figure 3C & 3D, over-expression of miR-145 led to a marked decrease in the expression of endogenous FLI-1 protein compared to OS cells transfected with control (U2OS and MG63 cell groups: both *P* < 0.001).

### miR-145 suppresses cell proliferation by targeting *FLI-1*

**Figure 4:**
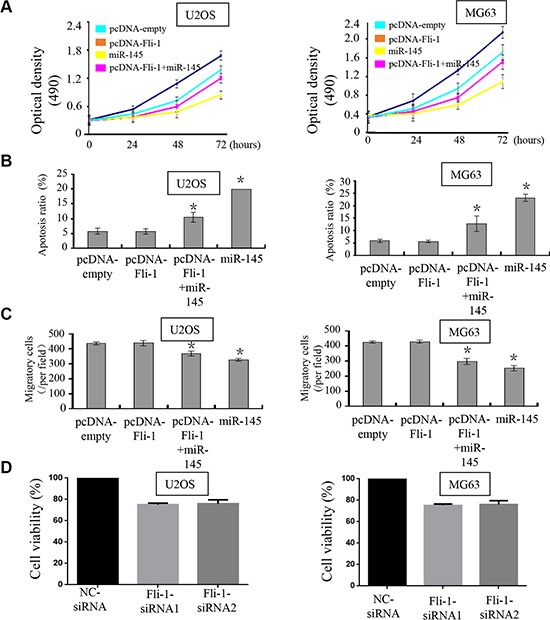
miR-145 suppresses tumor progression through targeting *FLI-1* Upon transfection with *FLI-1* construct, we rescued the expression of *FLI-1* in U2OS and MG-63 cells. The expression of *FLI-1* protein was validated by western blot assays; (**A**) CCK-8 assays were used to detect in U2OS and MG-63 cells co-transfected with miR-145 mimic and pcDNA-*FLI-1*-plasmid; (**B**) Cell apoptosis of U2OS and MG-63 cells treated as described was detected by Annexin V-PI staining; and (**C**) Transwell assays were performed to detect the effects on cell migration of U2OS and MG-63 cells treated as described. Upon transfection with the *FLI-1* plasmid, miR-145-mediated suppression of cell proliferation and cell migration was abolished, and promotion of cell apoptosis was inhibited. (×200); (**D**) We detected the effect of *FLI-1* siRNA in MG63 and U2OS cells by CCK8 assay. *p < 0.05 between two groups as indicated with a line.

To explore whether miR-145-mediated growth inhibition in OS cells via direct targeting of *FLI-1*, we performed a rescue experiment. We generated a new construct containing the full ORF of the *FLI-1* gene (pcDNA-*FLI-1*). As expected, *FLI-1* expression was rescued when pcDNA-*FLI-1* was transfected into U2OS and MG-63 cells that had been treated with miR-145 mimic for 24 h (Figure 4A). In agreement with the restored expression of *FLI-1*, increased cell proliferation (Figure 4B), accompanied by decreased cell apoptosis (Figure 4C) were also observed in OS cells transfected with pcDNA-*FLI-1* following treatment with miR-145. Moreover, upon transfection with the *FLI-1* construct, the suppression of miR-145-mediated cell migration (Figure 4A & 4B) in U2OS and MG-63 cells was also abolished. We also tested siRNA of *FLI-1* in OS cells and performed a proliferation assay. Knock-down of *FLI-1* expression by siRNA inhibited OS cell proliferation compared with the control siRNA (Figure 4D). Taken together, these results demonstrate that the repression of OS cell progression by miR-145 is a consequence of decreased *FLI-1* expression.

### miR-145 suppresses OS xenograft tumor growth and *FLI-1* expression *in vivo*

**Figure 5:**
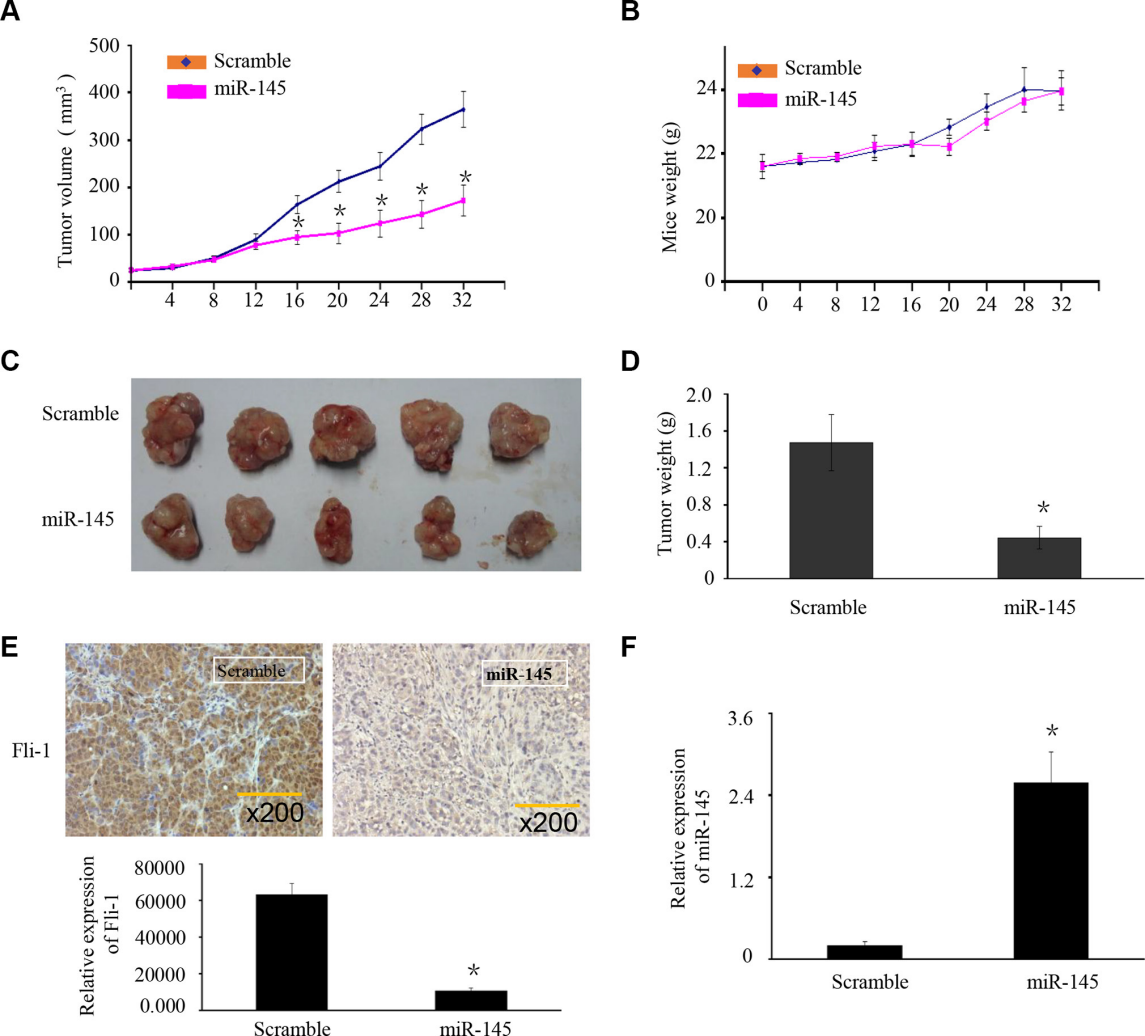
miR-145 suppresses OS xenograft tumor growth and *FLI-1* expression *in vivo* (**A**) U2OS cells stably over-expressing miR-145 or scrambled miRNA were subcutaneously injected into nude mice. Four weeks later, U2OS cells stably over-expressing miR-145 had smaller tumors than controls. (A) The growth curves of tumor volumes. (**B**) Average body weights. (**C**) Representative image of tumors formed. (**D**) Tumor weights. (**E**) Expression of FLI-1 in the zenograft tumor was detected by IHC. (**F**) Expression of miR-145 in the zenograft tumor was detected by RT-PCR. Data are presented as the mean ± SD. *P < 0.01 compared with control using the student's *t*-test.

To explore whether ectopic expression of miR-145 affects tumor growth *in vivo*, we performed a xenograft tumor model assay by subcutaneously injecting U2OS cells stably over-expressing miR-145 or scrambled miRNA in the dorsal flank of nude mice. The tumors in the group injected with U2OS cells stably over-expressing miR-145 grew at a slower rate and had smaller volumes than the scrambled control group (Figure 5A). The average bodyweight was not lower in the miR-145 over-expressing group than the scrambled control group (Figure 5D). To identify whether the expression of *miR-145* and FLI-1 were changed in the xenograft tumors we used RT-PCR and IHC, respectively. Compared with the scrambled miRNA group, the expression of *miR-145* was increased in the over-expressing group (Figure 5F). In contrast, the expression of FLI-1 was lower in the miR-145 over-expressing tumors (Figure 5E). These results suggested that miR-145 suppresses OS tumor growth and FLI-1 protein expression *in vivo*.

## DISCUSSION

In this study, we provide important evidence in support of miR-145 functioning as a tumor suppressor by targeting the expression of *FLI-1* in OS. First, we detected the expression of *miR-145* in 13 paired OS tissues and normal bone tissues. Our study validated the under-expression of *miR-145* in OS tissues and cell lines. Our results are consistent with the results of Xu et al, which found that *miR-145* is under-expressed in bladder cancer cell lines, and that over-expression of miR-145 inhibits cell proliferation by targeting *FSCN1* [13]. Along with our findings, these data suggest the tumor suppressor role of miR-145.

As *miR-145* expression is decreased in OS tissues and cell lines, we sought to compensate for its loss through exogenous transfection with miR-145 mimic into U2OS and MG-63 cells. Restored expression of miR-145 markedly inhibited cell proliferation and induced cell apoptosis. Moreover, miR-145-transfected cells showed a dramatic decrease in cell migration, accompanied with suppressed expression of *FLI-1*, which contains a putative binding sites of miR-145 in its 3′UTR identified by the prediction program MicroCosm Targets. Using luciferase assays, we confirmed the function of these binding sites; upon transfection with miR-145, the expression of *FLI-1* mRNA and protein were both suppressed. To our knowledge, this is the first observation of miR-145/*FLI-1* interaction in OS cells.

*FLI-1* acts as an oncogene [26, 27], and the etiology of a number of virally induced leukemias, as well as human Ewing's sarcoma, has been associated with *FLI-1* over-expression [28]. Aberrant expression of *FLI-1* has been recognized as a seminal event in the initiation of certain types of malignant transformation. *FLI-1* is also expressed at high levels in F-MuLV-induced erythroleukemias, malignant melanoma, small cell carcinomas of the lung and adenocarcinomas [29, 30]. Suppression of *FLI-1* inhibits cell proliferation and induces cell apoptosis of human cancer cell by targeting *Rb*, *GATA-1*, and *BCL-2* [31–33]. Anti-*FLI-1* compounds demonstrate strong anti-leukemic activity in a mouse erythroleukemia model that over-expresses *FLI-1*, making it possible to target *FLI-1* as a treatment strategy [34]. In this paper, we found that the activation of *FLI-1* might be a consequence of constitutive suppression of miR-145. Moreover, we restored the expression of *FLI-1* in miR-145-transfected cells to explore the role of *FLI-1* in miR-145-mediated tumor suppression. As expected, restoring the expression of *FLI-1* in OS cells abolished miR-145-mediated suppression, suggesting that *FLI-1* might have a key role in miR-145 mediated OS tumor suppression.

**Figure 6:**
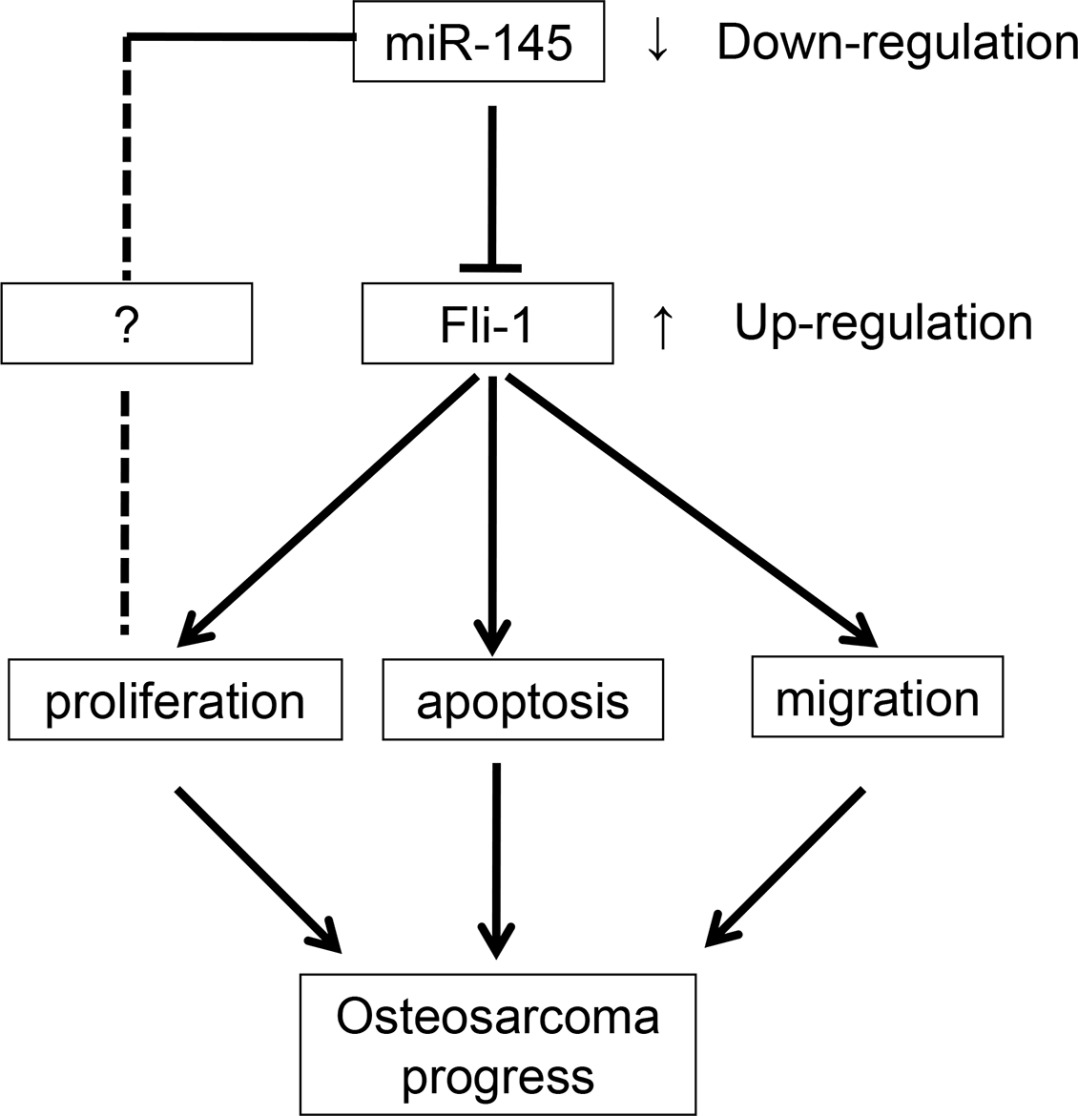
Summary of the signaling pathways regulated by miR-145 and directly influencing OS cell proliferation, apoptosis, and migration

In summary (Figure 6), this study identified *FLI-1* as a novel gene target of miR-145 in OS. These results demonstrate that miR-145 acts as a tumor suppressor, targeting the 3′-UTR of *FLI-1* mRNA. Down-regulation of *FLI-1* has a profound effect on the growth and migration of OS cells. Its inhibition of growth in OS cells is compatible with the well-known ability of *FLI-1* to stimulate cell proliferation. miR-145 regulates *FLI-1* at the both the levels of protein and mRNA, although the specific mechanism requires further investigation. These studies extend our knowledge concerning *FLI-1* as an oncogene involved in OS tumorigenesis.

## MATERIALS AND METHODS

### Specimens

Thirteen fresh specimens of osteosarcoma and matched normal tissues were collected from the Department of Orthopedics, Xiang Ya Hospital of the Central South University. The matched normal tissues were obtained 5 cm distant from the tumor margin, which were confirmed by pathologists. No patients underwent any therapy before study recruitment. Use of the tissue samples and all experiments were approved by the Institutional Ethics Committee.

### Cell lines and cell culture

Human osteosarcoma cell lines (HOS, Saos-2, U2OS and MG-63) or normal osteoblast cells (NHOst) were obtained from the American Type Culture Collection (ATCC, Manassas, VA, USA). Cells were maintained in Dulbecco's modified Eagle medium (DMEM, Gibco, Life Technologies, Darmstadt, Germany), supplemented with 10% fetal bovine serum (FBS; PAA, Pasching, Austria), streptomycin (100 μg/mL), and penicillin (100 U/mL). Cultures were incubated in a humidified atmosphere of 5% CO_2_ at 37°C.

### Reverse transcription and quantitative real-time PCR

Total RNA was extracted from cultured cells, fresh OS tissues, and xenograft mouse tumors with Trizol reagent (Ambion) according to the manufacturer's protocol. miRNA cDNA was synthesized using a One-Step PrimeScript miRNA cDNA Synthesis Kit (Takara). Quantitative real-time PCR (qRT-PCR) was performed with SYBR green Premix Ex Taq II (Takara) with a StepOne Plus Real-Time PCR System (Applied Biosystems). Expression of *U6* was used as an endogenous control. Specific primers qualified with a probe for reverse transcription were used as follows: *MIR-145*: 5′-GTCCAGTTTTCCCAGGAATCCCT-3′; and *U6*: 5′-AACGCTTCACGAATTTGCGT-3′. The expression of *GAPDH* was used as an internal control and Oligo(dT) was used as the primer for *FLI-1* and *GAPDH* reverse transcription. Then, qRT-PCR was performed to quantify relative expression of *FLI-1* with the following primers:

*FLI-1*: sense, 5′-CAGTCGCCTAGCCAACCCTG and antisense, 5′-GCAATGCCG TGGAAGTCAAAT. *GAPDH*: sense, 5′-TCAACGACCACTTTGTCAAGC TCA-3′ and anti-sense, 5′-GCTGGTG GTCCAGGGGTC TTACT-3′. PCR efficiencies were calculated with a relative standard curve derived from a complementary DNA mixture that gave regression coefficients > 0.95. The thermal profile for the qPCR was 95°C for 1 min, followed by 35 cycles of 93°C for 15 sec, 62°C for 30 sec and 72°C for 10 sec on a Bio-Rad CFX96 qRT-PCR system (Bio-Rad, Hercules, CA, USA). All qPCR experiments were performed in triplicate.

### Cell transfection and reporter gene analysis

miR-145 mimic and scramble mimic were purchased from Dharmacon (Austin, TX, USA). All oligonucleotides were transiently transfected into U2OS and MG-63 cells lines with a final concentration of 50 nM using Lipofectamine 3000 Reagent (Invitrogen, Carlsbad, CA) following the manufacturer's protocol. Briefly, the cells were seeded in 6-well, 24-well, or 96 well plates at 50% confluence the day before transient transfection into the OS cell lines. miR-145 precursor, miR-145 inhibitor, and microRNA control (50 nM each), were used for each transfection.

### Plasmid construction and primer sequences

The full-length *FLI-1* gene open reading frame (ORF) was amplified by PCR and cloned into a pCDNA3.1 construct to generate the pCDNA3.1-*FLI-1* construct. The empty pCDNA3.1 construct was used as a control. Primer sequences used for PCR were, sense: 5′-AAACGAGGGAAATGGGAG-3′ and anti-sense: 5′-TACCAACGGTGTCAACCTG-3′. Following bioinformatic analysis, the putative microRNA regulatory element (MRE, Figure 3A) of miR-145 was chemically synthesized and cloned into a pGL3 control vector (Promega) at the Xba1 restriction site. To construct the Luc-*FLI-1*-3′UTR-Full vector, the full length 3′UTR of *FLI-1* was amplified from the cDNA of U2OS cells using *FLI-1* PCR primers; sense: 5′CTAGAGAAGCCCATCCTGCACACT 3′ and antisense: 5′CTAGACGTTGTTTTTCCCAGAGCT 3′, and then cloned into the pGL3 control vector at the Xba1 site. To create the mutated *FLI-1*-3′UTR vector, eight nucleotides (TGGGATTCCTGGGAATTAACCTG) were changed for the reporter construct. All of the constructs were designed and synthesized by Cyagen Biosciences, Inc.

### Proliferation assay

Before analysis of cell proliferation, U2OS and MG-63 cells were seeded into 96-well plates at a concentration of 8 × 10^3^ cells/well. The Cell Counting Kit-8 (CCK-8, Dojindo, Kumamoto, Japan) was added to the wells at 0, 24, 48, and 72 hours post-transfection, and cells were diluted in normal culture medium at 37°C. The absorbance values of optical density (OD) in each well were measured with a microplate reader set at 490 nM (OD490). All experiments were performed three times and the average percentages of cells shown.

### Apoptosis assay

Cells were transfected with miR-145 mimics or control mimics for 24 h. Forty-eight hours after transient transfection, according to the Annexin V-PI Apoptosis Detection Kit I (BD Pharmingen, CA, USA) protocol, cells were harvested and re-suspended with 500 μL of binding buffer. The cell suspension was incubated with 5 μL annexin-V-FITC and propidium iodide buffer at room temperature for 20 min. The stained cells were analyzed on a Flow Cytometer (BD Biosciences, NJ) and apoptotic cells were quantified by apoptosis ratio. The experiment was repeated three times.

### Migration assays

Migration assays were carried out in modified Boyden chambers (BD Biosciences, San Jose, CA, USA) with 8 μm pore filter inserts in 24-well plates. Twenty-four hours after transfection, 3 × 10^5^ cells suspended in serum-free DMEM were added to the upper chamber. DMEM containing 20% FBS was added to the lower chambers as a chemoattractant. Twenty-four hours later, the non-filtered cells were gently removed with a cotton swab. Filtered cells located on the lower side of the chamber were stained with crystal violet, air-dried, and photographed. The transmembrane cells in each treatment group were counted. Three independent experiments were performed.

### Luciferase activity assays

The entire 3′-UTR of the *FLI-1* gene was cloned into the pGL-3-vector (Promega, WI, USA) immediately downstream of the Renilla luciferase gene. Mutations in the 3′-UTR of the *FLI-1* gene with miR-145 target sites deleted (Mut-1) were generated with the QuickChange Site-Directed Mutagenesis kit (Stratagene, CA, USA). Approximately 1 × 10^5^ MG-63 cells per well were seeded into 24-well plates for 24 h before transfection. Cells were co-transfected with 50 ng pGL-3 firefly luciferase reporter, 10 ng pRL-TK Renilla luciferase reporter, and 50 nM miR-145 mimics or scramble mimics using Lipofectamine 2000 (Invitrogen, Carlsbad, CA, USA). A luciferase reporter construct containing the miR-145 consensus target sequence served as a positive control and the pRL-TK vector served as an internal control. Cell lysates were prepared using Passive Lysis Buffer (Promega, Madison, WI, USA) 48 h after transfection, and luciferase activity was measured using the Dual-Luciferase Reporter Assay (Promega, Madison, WI, USA). Results were normalized to the Renilla luciferase, and the scramble groups as the standard, which was divided by the treatment groups. Experiments were independently repeated three times.

### Western blot analysis

Cells were lysed in RIPA buffer. Cellular proteins were collected and subjected to 10% SDS-PAGE, and transferred onto PVDF membranes (Amersham Pharmacia Biotech, Piscataway, NJ). The membranes were then incubated with specific primary antibodies. Anti-FLI-1 was purchased from Santa Cruz Biotechnology, Inc. Anti-β-ACTIN was purchased from Epitomics, Inc. This was followed by incubation with horseradish peroxidase-conjugated anti-rabbit or anti-mouse IgG antibodies from Santa Cruz Biotechnology, and antigen-antibody complexes were visualized using the Western Bright ECL detection system (Advansta, CA).

### Immunohistochemistry

Immunohistochemistry was used to detect FLI-1 expression in xenograft mouse model tumors. Sections (4 μm) were cut from formalin-fixed paraffin-embedded tissue blocks and then deparaffinized in xylene and rehydrated in successive washes of ethanol. The sections were then heated in a microwave oven at medium power for 2 minutes in citrate buffer (pH 6.0) for heat-induced epitope retrieval. The sections were subjected to blockade of endogenous peroxidase activity and nonspecific binding of the primary antibody, and then target protein localization with the first antibody and visualization with the secondary antibody and color reaction as described above. Subsequently, the slides were incubated with rabbit monoclonal antibody anti-FLI-1 (1:100 dilution), for overnight at 4°C in a moist chamber. The slides were sequentially incubated with a secondary antibody for 1 hour at room temperature, and stained with 3,3-diaminobenzidine. Finally, the sections were counterstained with Mayer's hematoxylin, dehydrated, and mounted. A negative control was obtained by replacing the primary antibody with PBS.

### Animal experiments

To determine the effect of miR-145 on OS tumor growth in a xenograft model, six-week old athymic Nu/nu nude mice were maintained in a pathogen-free environment. Five NC and five miR-145 mice groups were used (for a total of 10 mice). In brief, U2OS cells (2 × 10^6^) were inoculated subcutaneously into the flank of the nude mice. Once palpable tumors developed (average volume 50 mm^3^), mice were treated with 100 nM synthetic miR-145 complexed with 100 μl transfection reagent in 50 μl PBS delivered nine times intratumorally every three days. Tumor growth was followed for 32 days from first injection until tumors reached about 400 mm^3^ total volume, at which time mice were euthanized. Tumor volume was calculated using the following formula: V = (width^2^ x length)/2. Body weights were also recorded. Primary analyses involved the comparison (for each time point separately) between the NC group and the miR-145 group. Animal experiments were approved by the Animal Research Committee of Central South University and were performed in accordance with established guidelines.

### Statistical analysis

All data are expressed as the mean ± standard deviation of at least three independent experiments. Statistical analysis was carried out using SPSS 18.0 software (SPSS Inc.; Chicago, IL, USA). *p*-values < 0.05 were considered significant.
